# Prediction of the Mechanism of Action of Fusaricidin on *Bacillus subtilis*

**DOI:** 10.1371/journal.pone.0050003

**Published:** 2012-11-21

**Authors:** Wen-Bang Yu, Chun-Yun Yin, Ying Zhou, Bang-Ce Ye

**Affiliations:** Lab of Biosystems and Microanalysis, State Key Laboratory of Bioreactor Engineering, East China University of Science and Technology, Shanghai, China; Cinvestav, Mexico

## Abstract

Long-term use of antibiotics has engendered a large number of resistant pathogens, which pose a serious threat to human health. Here, we investigated the mechanism of fusaricidin antibacterial activity toward *Bacillus subtilis* and characterized the pathways responsible for drug resistance. We found that σ^w^, an extracytoplasmic function sigma factor, plays an important role in the resistance to fusaricidins during the initial 5 minutes of drug addition. Approximately 18 genes were induced more than 3-fold, of which 66.7% are known to be regulated by σ^w^. Over the following 3 h, fusaricidins induced 194 genes more than three-fold, and most were associated with classes of antibiotic-responsive stimulons. Moreover, the fusaricidin treatment increased the catabolism of fatty and amino acids but strongly repressed glucose decomposition and gluconeogenesis. In summary, our data provide insight into the mechanism of fusaricidin activity, on which we based our suggested strategies for the development of novel antibiotic agents.

## Introduction

The emergence of rapid resistance to antibiotics among major pathogens remains a serious threat to public health. To overcome this challenge, many laboratories pursue drug discovery efforts to identify novel structural classes of antibiotics [1]. However, a limited understanding of the molecular mechanism of action (MoA) is a critical bottleneck in the development of novel classes of antibacterial agents. Therefore, more detailed studies into the mechanism of microbial death resulting from antibiotic use and the reason for the drug resistance of pathogens is required.

Antibacterial peptides, a cluster of small peptides secreted by most organisms, represent a promising new class of antibiotic drugs. They are known to be active against a wide range of microorganisms including bacteria, protozoa, yeast fungi, viruses, and even tumor cells. These active polypeptides have characteristics of small molecular mass, high efficacy, stability, particular antibacterial mechanism, and little drug resistance. Fusaricidin A was elucidated to be a cyclic depsipeptide containing a unique fatty acid, 15-guanidino-3-hydroxypentadecanoic acid. Fusaricidins B, C, and D are minor components from the culture broth of a bacterial strain *Bacillus polymyxa* KT-8. Their structures have been elucidated to be cyclic hexadepsipeptide, very similar to that of fusaricidin A. Fusaricidins C and D displayed strong activity against gram-positive bacteria, especially *Staphylococcus aureus* FDA 209P, *S. aureus*, and *Micrococcus luteus* IFO 3333 as did fusaricidin A, whereas fusaricidin B showed weaker activity against those microbes than the fusaricidin C and D mixture. However, fusaricidin, even at 100 µg/mL, showed no activity against all the gram-negative bacteria tested [2], [3].

Despite their promising antimicrobial profile, much remains to be determined regarding the MoA of fusaricidins and the development of microbial resistance to the compounds. In this report, we used genome-wide expression technologies to elucidate bacterial defense mechanisms responsible for fusaricidin resistance; this strategy is increasingly used in the antibiotic research field [4], [5]. As a model organism, we chose *B. subtilis* 168, a gram-positive, spore-forming bacterium that is ubiquitously distributed in soil. The complete genome of *B. subtilis* 168 was sequenced in 1997 and is reported to encode 4,106 proteins [6]. The availability of this genomic sequence provides a cost-effective opportunity to explore genomic variation between strains.

**Figure 1:**
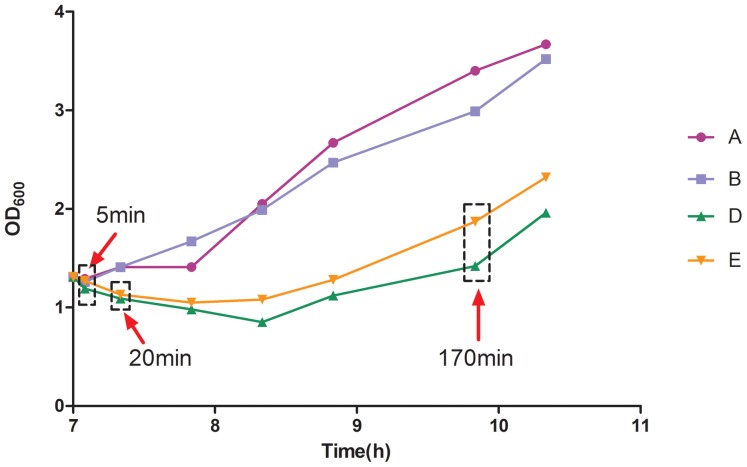
Time points of the transcriptome experiments. A and B are duplicate control samples; D and E are duplicate samples treated with fusaricidin after the 7-h culture of *B. subtilis* 168.

Trancriptomic analysis is a powerful approach to elucidate the inhibitory mechanisms of novel antimicrobial compounds and has been successfully applied to characterize and differentiate antimicrobial actions, often using *B. subtilis* as a model organism [7], [8]. In this report, we combined transcriptomic analyses with studies of the genetic and physiological responses of *B. subtilis* to fusaricidins. The profiling revealed that fusaricidins strongly activated SigA, a protein that regulates RNA polymerase to control cell growth. Kinetic analyses of transcriptional responses showed that differentially regulated genes represent several metabolic pathways, including those regulating proline levels, ion transport, amino acid transport, and nucleotide metabolism.

## Materials and Methods

### Bacterial Strain and Media

*B. subtilis* 168 was stored in our laboratory. LB (Luria-Bertani) medium (10-g tryptone, 5-g yeast extract, and 10-g NaCl per liter of distilled H_2_O) was used to grow *B. subtilis* cultures.

### Growth Conditions

In our experiments, *B. subtilis* 168 was used, stored at −20°C in 25% glycerol. It was inoculated in LB medium and grown overnight at 37°C and 200 rpm. Then, the seed culture was used to inoculate 10 mL of fresh LB medium. To study the effect of fusaricidin on *B. subtilis* 168 cells and the corresponding transcriptomic profiles, fusaricidin (1.713 µg/mL) was added at an OD_600_ of approximately 1.30 at the exponential growth phase (7-h culture period). Two independently cultured replicates were performed, respectively. Samples were taken to measure the OD_600_ at designated time points (5, 20, and 170 min) and to extract RNA for the following experiments.

**Figure 2:**
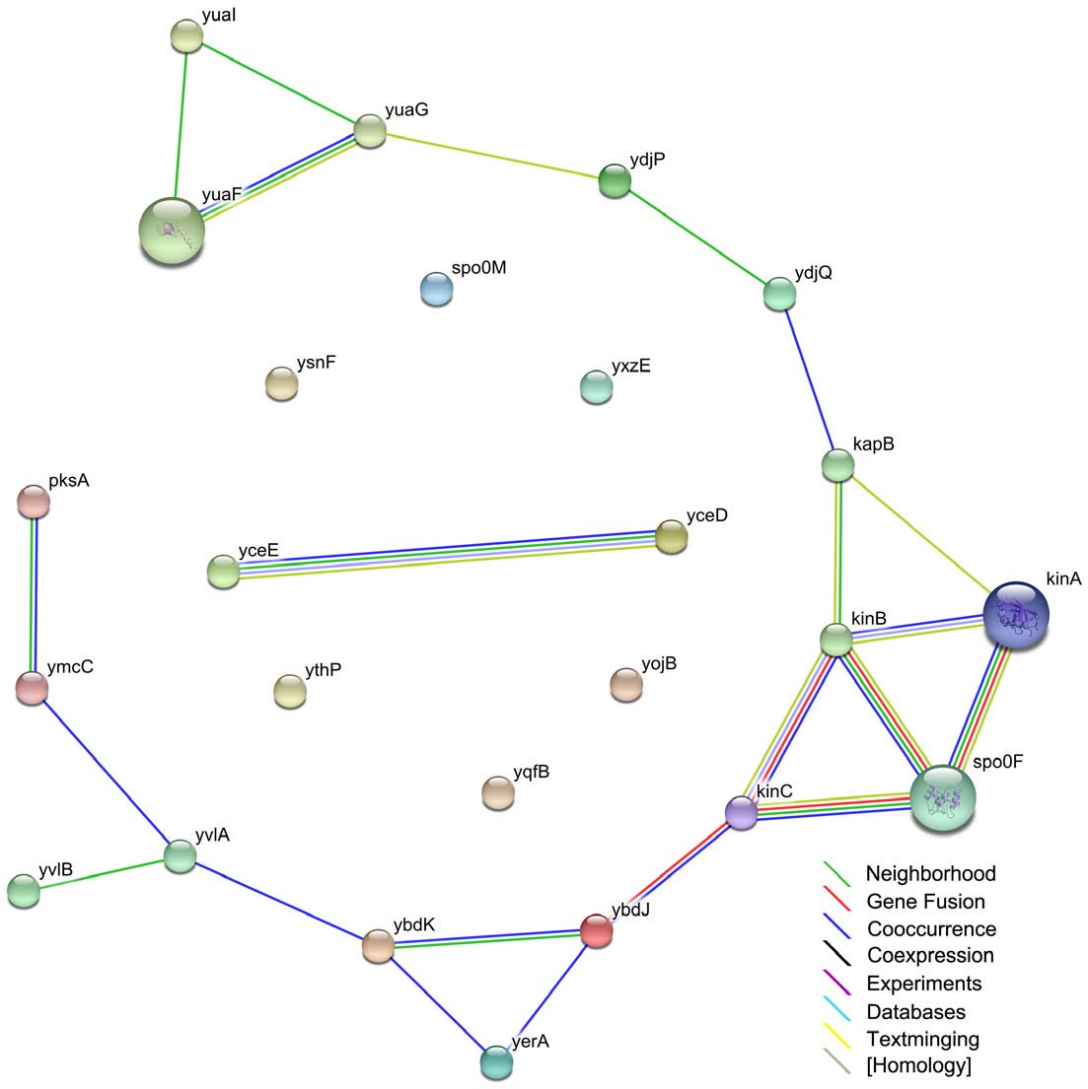
Protein-protein interaction networks at 5 min using the string analysis.

**Figure 3:**
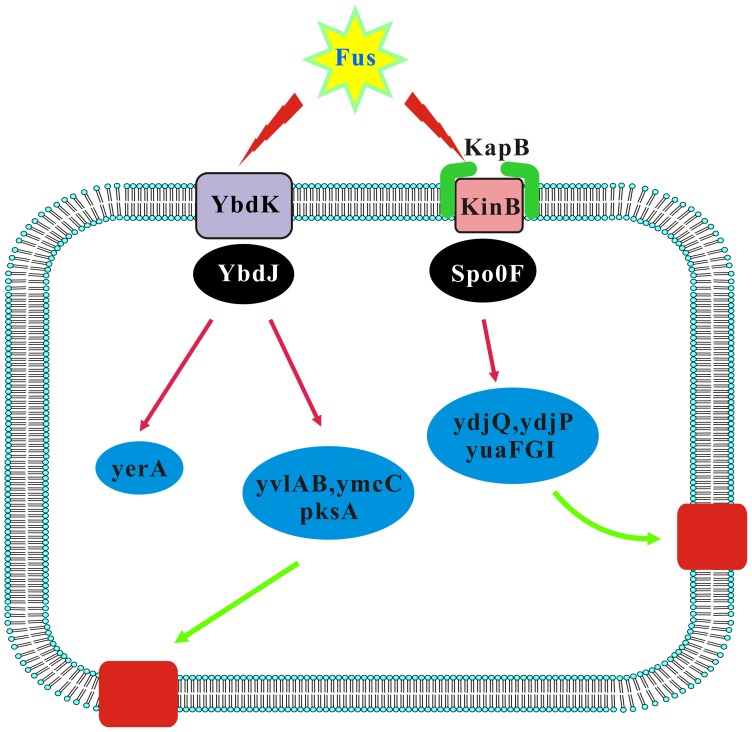
The rapid-response pathways of *B. subtilis* to the fusaricidin treatment. Fus, fusaricidin. The red columns indicate the hypothetical proteins translated from the genes in the corresponding blue ellipses.

### RNA Preparation and Microarray Analyses

The cultures were grown to mid-log phase (OD_600_ of 1.30) and split into 2 flasks. One culture was treated with fusaricidin (1.6 µg/mL), and the other was untreated as a control. In parallel, 2 independent array experiments from separate cultures with fusaricidin treatment were performed with biological duplicates. The *B. subtilis* cells were harvested at 5, 20, and 170 min after fusaricidin addition. RNA isolation and microarray analysis were performed as previously described for amino acid addition [9].

**Figure 4:**
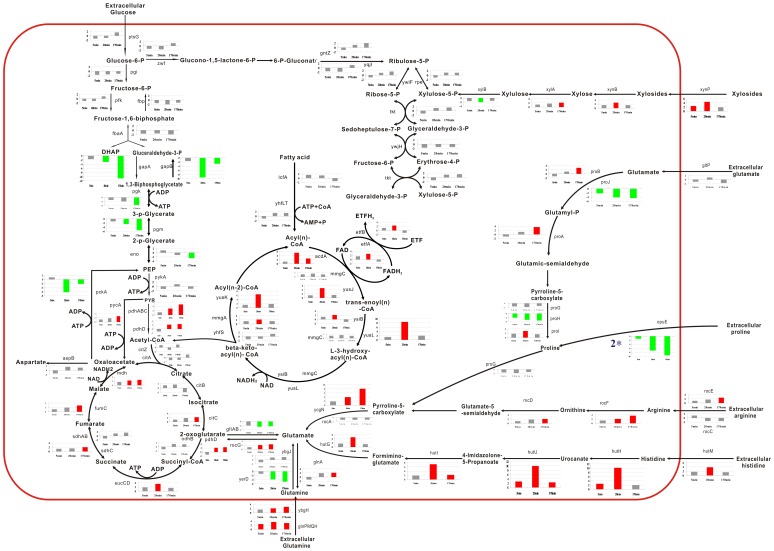
The metabolic changes of carbon and nitrogen. The expression of genes related to the central carbon and nitrogen pathways are schematically presented. The 3 bars from left to right represent the fold changes of the gene expressions in response to the 3 time points (5, 20, and 170 min). The red bars represent an upregulation; the green bars, a downregulation; and the gray bars, the messages that did not significantly change relative to our cutoff (3-fold increase in expression).

All the microarray data reported in the manuscript are described in accordance with the MIAME (Minimum Information About a Microarray Experiment) guidelines. A 3-fold change was used as the threshold for selection of fusaricidin-induced genes.

### MISP Analysis

The differentially expressed genes chosen with a coefficient of variation >0.1 were distributed over the MIPS functional categories for their classification (http://mips.gsf.de/projects/).

## Results and Discussion

### *B. subtilis* 168 Cell Growth was Inhibited by Fusaricidin

Changes in cell growth (measured as change in cell concentration) were studied for 3.5 h after the addition of fusaricidin (<1 minimal inhibitory concentration [MIC], 1.713 µg/mL). As shown in Figure 1, the replicates of the cells treated with fusaricidin grew more slowly; by contrast, the replication of the control was continuous. This indicates that fusaricidin is toxic to *B. subtilis* 168.

**Table 1:** The MIPS analysis of the differential genes at 20 min.

FUNCTIONAL CATEGORY	P VALUE
01.01.03.03 metabolism of proline	7.69E-03
01.01.03.03.01 biosynthesis of proline	5.21E-03
01.01.09.07 metabolism of histidine	1.94E-02
01.01.09.07.01 biosynthesis of histidine	1.94E-02
01.03 nucleotide/nucleoside/nucleobase metabolism	3.01E-03
01.03.01 purine nucleotide/nucleoside/nucleobase metabolism	1.52E-03
01.03.01.03 purine nucleotide/nucleoside/nucleobase anabolism	1.34E-07
01.03.04 pyrimidine nucleotide/nucleoside/nucleobase metabolism	2.98E-02
02.25 oxidation of fatty acids	7.69E-03
20 CELLULAR TRANSPORT, TRANSPORT FACILITIES, AND TRANSPORT ROUTES	9.41E-04
20.01 transported compounds (substrates)	1.19E-05
20.01.01 ion transport	4.51E-03
20.01.01.01 cation transport (H+, Na+, K+, Ca2+, NH4+, etc.)	1.22E-03
20.01.01.01.01 heavy metal ion transport (Cu+, Fe3+, etc.)	1.62E-02
20.01.07 amino acid/amino acid derivatives transport	2.73E-02
20.01.17 nucleotide/nucleoside/nucleobase transport	2.77E-02
20.01.27 drug/toxin transport	1.97E-02
20.03 transport facilities	1.98E-02
20.03.02 carrier (electrochemical potential-driven transport)	7.36E-04
20.03.02.02 symporter	5.60E-04
20.03.02.02.01 proton driven symporter	7.85E-03
20.03.02.02.02 sodium driven symporter	1.63E-02
20.09 transport routes	3.51E-04
20.09.18 cellular import	7.13E-04
32 CELL RESCUE, DEFENSE, AND VIRULENCE	6.00E-04
32.01 stress response	7.49E-06
32.01.01 oxidative stress response	4.62E-03
32.07 detoxification	8.33E-04
32.07.05 detoxification by export	1.55E-02
32.07.07 oxygen and radical detoxification	1.61E-04
32.07.07.01 catalase reaction	1.72E-02
34 INTERACTION WITH THE ENVIRONMENT	4.61E-02
34.01 homeostasis	1.12E-03
34.01.01 homeostasis of cations	3.22E-04
70.30 prokaryotic cytoplasmic membrane	1.40E-02

**Figure 5:**
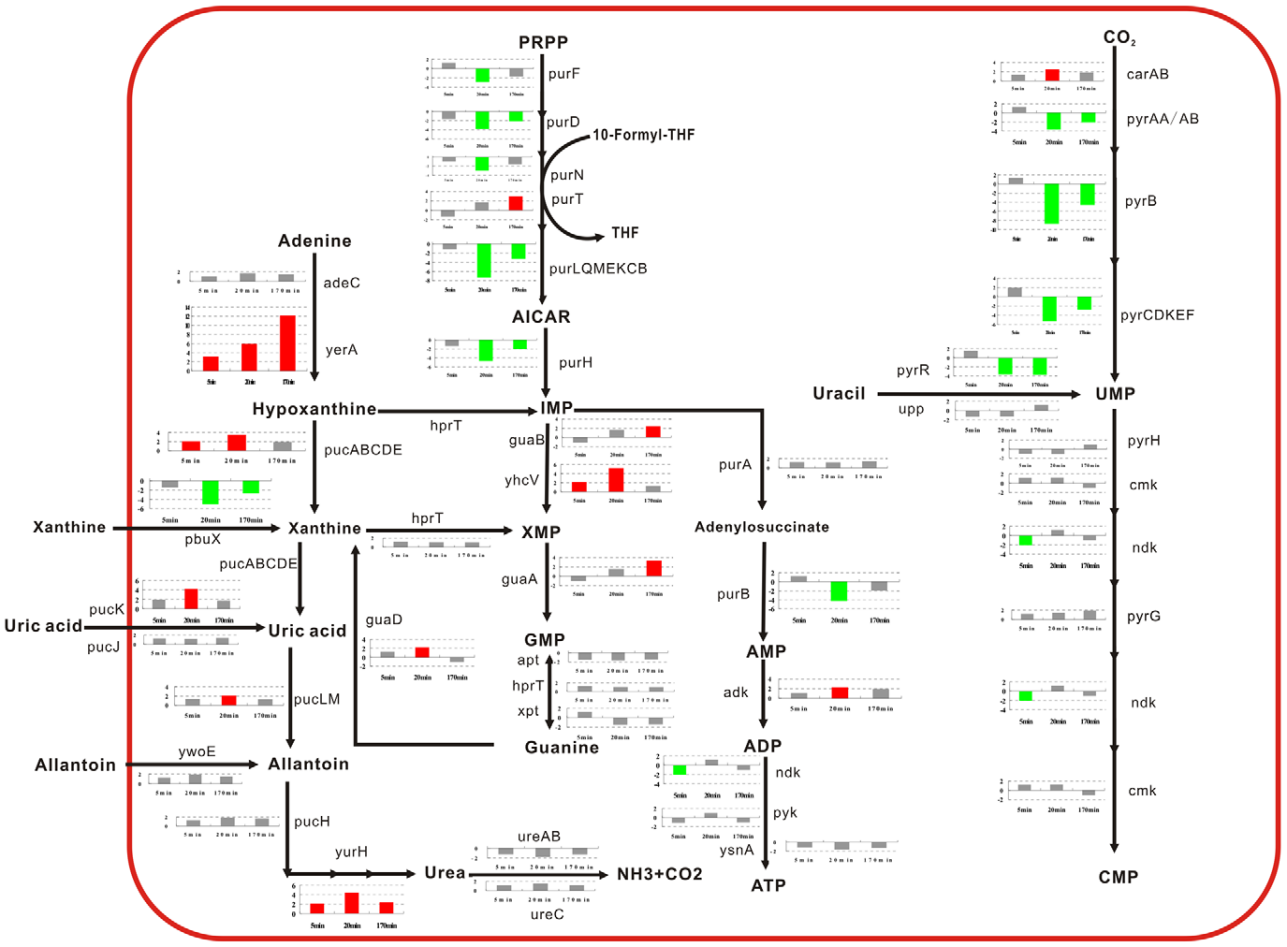
Changes in nucleotide metabolism. The expression of genes related to nucleotide metabolism are schematically presented. The 3 bars from left to right represent the fold changes of the gene expressions in response to the 3 time points (5, 20, and 170 min). The red bars represent an upregulation; the green bars, a downregulation; and the gray bars, the messages that did not significantly change relative to our cutoff (3-fold increase in expression).

The influence of fusaricidin on the transcriptome of logarithmically growing *B. subtilis* cells was quantified using fluorescent DNA microarray technology. Changes in gene expression were studied by the addition of fusaricidin. Samples were taken at 5, 20, and 170 min after the addition of fusaricidin and compared with an untreated control sample taken at 5 min. When a 3-fold change (p value log ratio <0.05) relative to the control was used as a cutoff value, 18, 415, and 415 genes (approximately 0.44%, 10.11%, and 10.11% of all *B. subtilis* genes, respectively) were identified as significantly induced by fusaricidin at the respective time points.

**Table 2:** Gene groups with *E* <0.05 at 5, 20, and 170 min.

Time point of fermentation: 5 min				
Gene groups	*t* value	*E* value	Mean	ORFs
**SigW**	15.46	<10E-15	0.978	63
**CcpA-negative**	7.12	1.50E-09	0.376	120
**SigK**	6.50	1.12E-07	0.390	93
**SigE**	4.99	8.38E-04	0.263	148
**AbrB-negative**	4.94	1.09E-03	0.374	61
**AbrB-positive**	4.80	2.20E-03	0.592	21
**GerE-negative**	4.64	4.83E-03	0.847	10
**FNR-positive**	4.47	1.08E-02	0.871	9
**SigB**	−8.07	9.25E-13	−0.307	100
**Time point of fermentation: 20 min**				
**Gene groups**	***t*** ** value**	**E value**	**Mean**	**ORFs**
**CcpA-negative**	7.09	1.86E-09	0.737	120
**SigK**	6.34	3.19E-07	0.750	93
**AbrB-positive**	5.86	6.43E-06	1.380	21
**StrCon-negative**	5.48	5.91E-05	0.672	92
**SigE**	5.22	2.49E-04	0.537	148
**SigH**	−4.37	1.71E-02	−0.587	32
**StrCon-positive**	−4.62	5.32E-03	−0.414	59
**PyrR-negative**	−4.86	1.63E-03	−1.547	9
**CtsR-negative**	−5.93	4.21E-06	−1.579	12
**PurR-negative**	−8.76	<1.0E-15	1.314	32
**SigB**	−25.00	<1.0E-15	−2.003	100
**Time point of fermentation: 170 min**				
**Gene groups**	***t*** ** value**	***E*** ** value**	**Mean**	**ORFs**
**SigD**	12.98	<1.0E-15	1.588	77
**Fur-negative**	10.43	<1.0E-15	1.907	36
**SigA**	8.77	<1.0E-15	0.376	674
**Ccp-negative**	8.08	9.25E-13	0.820	120
**CodY-negative**	5.97	3.29E-06	1.016	44
**StrCon-negative**	5.66	2.10E-05	0.673	92
**SinR-negative**	5.50	5.28E-05	1.377	21
**lolR-negative**	4.75	2.82E-03	1.556	13
**AbrB-positive**	4.37	1.71E-02	1.106	21
**SigL**	4.18	3.97E-02	1.011	23
**AbrB-negative**	−4.79	2.31E-03	−0.597	61
**Rok-negative**	−5.85	6.83E-06	−1.124	29
**SigB**	−24.33	<1.0E-15	−2.339	100

### Fusaricidin Rapidly Induced σ^W^ Regulon in *B. subtilis*

In this study, DNA microarrays were used to investigate the global transcriptional response to fusaricidin. Approximately 18 genes were found to be induced by 3-fold during the first 5 min of the fusaricidin treatment, including *yqfB* (3.1-fold), which codes for a hypothetical protein; sporulation-control gene *spo0M* (6.5-fold); *pksA* (6.7-fold), which codes for a transcriptional regulator of polyketide synthase; and *yceD* (3.7-fold), which is similar to tellurium resistance protein. Two thirds (12/18) of the genes were identified as σ^W^ responsive. However, no significantly different expression was found after 20 min of treatment, indicating that the induction of these genes was rapid and transient. Only 1 gene, *ysnF* (coding for a protein with unknown function), which is controlled by the general stress σ^B^ factor, was repressed (2.5 fold) at 5 min post treatment. These observations suggest that fusaricidin rapidly induces a σ^W^ regulon response upon membrane damage. It is interesting that the fusaricidin treatment had no effect on the expression of the regulons controlled by other ECF sigma factors and the cell wall stress-related TCS systems (LiaRS, BceRS, PsdRS, YxdKJ, and YycFG).

**Figure 6:**
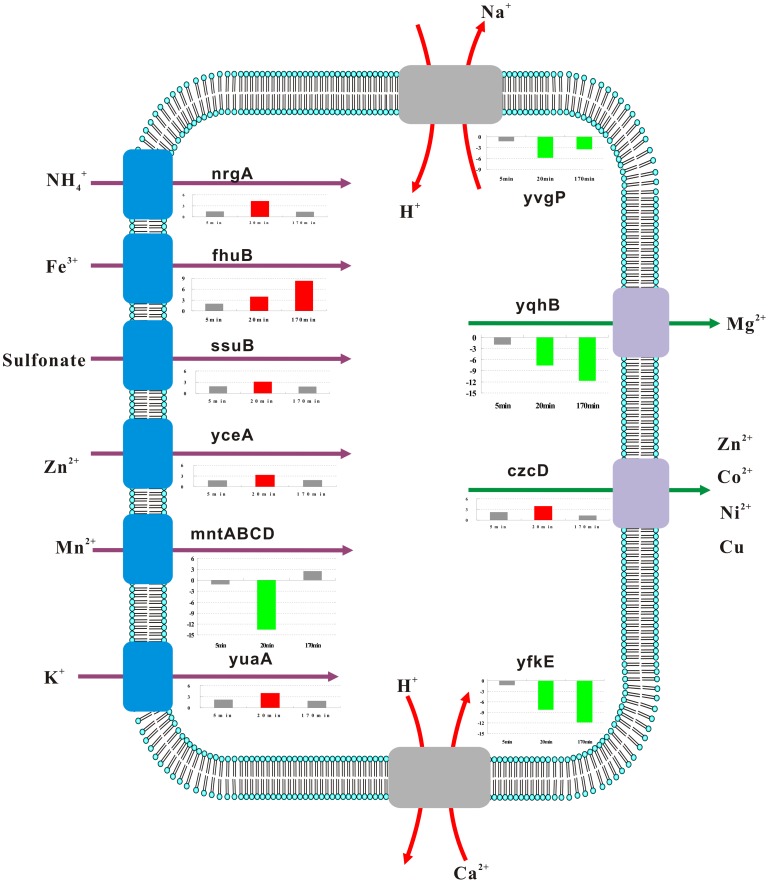
The transport of cations. The 3 bars from left to right represent the fold changes of the gene expressions in response to the 3 time points (5, 20, and 170 min). The red bars represent an upregulation; the green bars, a downregulation; and the gray bars, the messages that did not significantly change relative to our cutoff (3-fold increase in expression).

The strongest response to fusaricidin treatment was the induction of the *yuaFGI* operon (9.3- to 29-fold) and *ymcC* gene (approximately 17.6-fold). The *yuaFGI* operon contains 3 genes: *yuaF* (coding for membrane integrity integral inner membrane protein), *yuaG* (coding for flotillin-like protein), and *yuaI* (coding for acetyl-transferase, EC:2.3.1). The *yuaFGI* operon is also strongly induced by vancomycin [4] and the cationic antimicrobial peptide phosphatidylglycerol-1 (PG-1) [10]. *yuaG* is associated with negatively charged phospholipids, for example, PG or cardiolipin [11]. The gene *ymcC*, which encodes a transmembrane protein, is currently annotated as a hypothetical protein in the Subtilist and KEGG databases. A blastp homology search revealed that the *ymcC* gene was highly conserved in various species such as *Bacillus* and *Paenibacillus* species. The gene cluster (*fus* cluster) for the fusaricidin biosynthetic pathway has been identified and characterized in *Paenibacillus polymyxa* PKB1 [12]. It is intriguing that upstream of this cluster is a 531-bp ORF encoding a putative protein of 177 amino acids; this protein exhibits greatest similarity to *ymcC*. The gene *ymcC* of *B. subtilis* also precedes a cluster of putative polyketide synthase genes. Taken together, these findings suggest that the membrane protein YmcC, which is regulated by the σ^W^ factor, may play a role in the action of antibiotics on bacteria.

**Figure 7:**
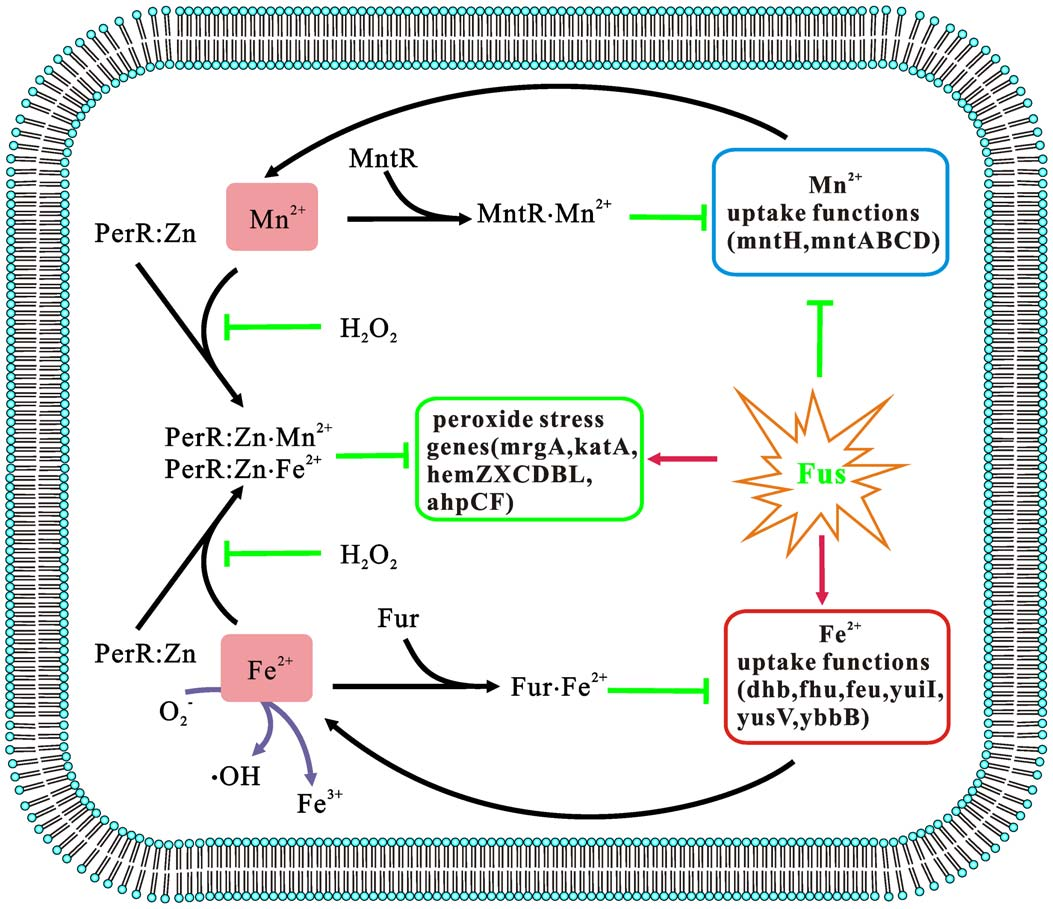
The transport and oxidation stress response associated with Fe^2+^ and Mn^2+^. Fus, fusaricidin.

The BacLight kit from Molecular Probes, Inc. (Eugene, Oreg.) was also used to examine fusaricidin-dependent membrane damage, as described by Hilliard [13]. In our previous study, cell membrane integrity damage was observed with *B. subtilis* 168 by fusaricidins at 4× MIC, whereas no damage was observed with the drug-free control. We subsequently confirmed using 2 independent assays (BacLight assay and transcriptome profiling) and various antibiotic concentrations (0.6 to 4× MIC) at which the MoA of fusaricidin is likely to involve membrane damage.

**Figure 8:**
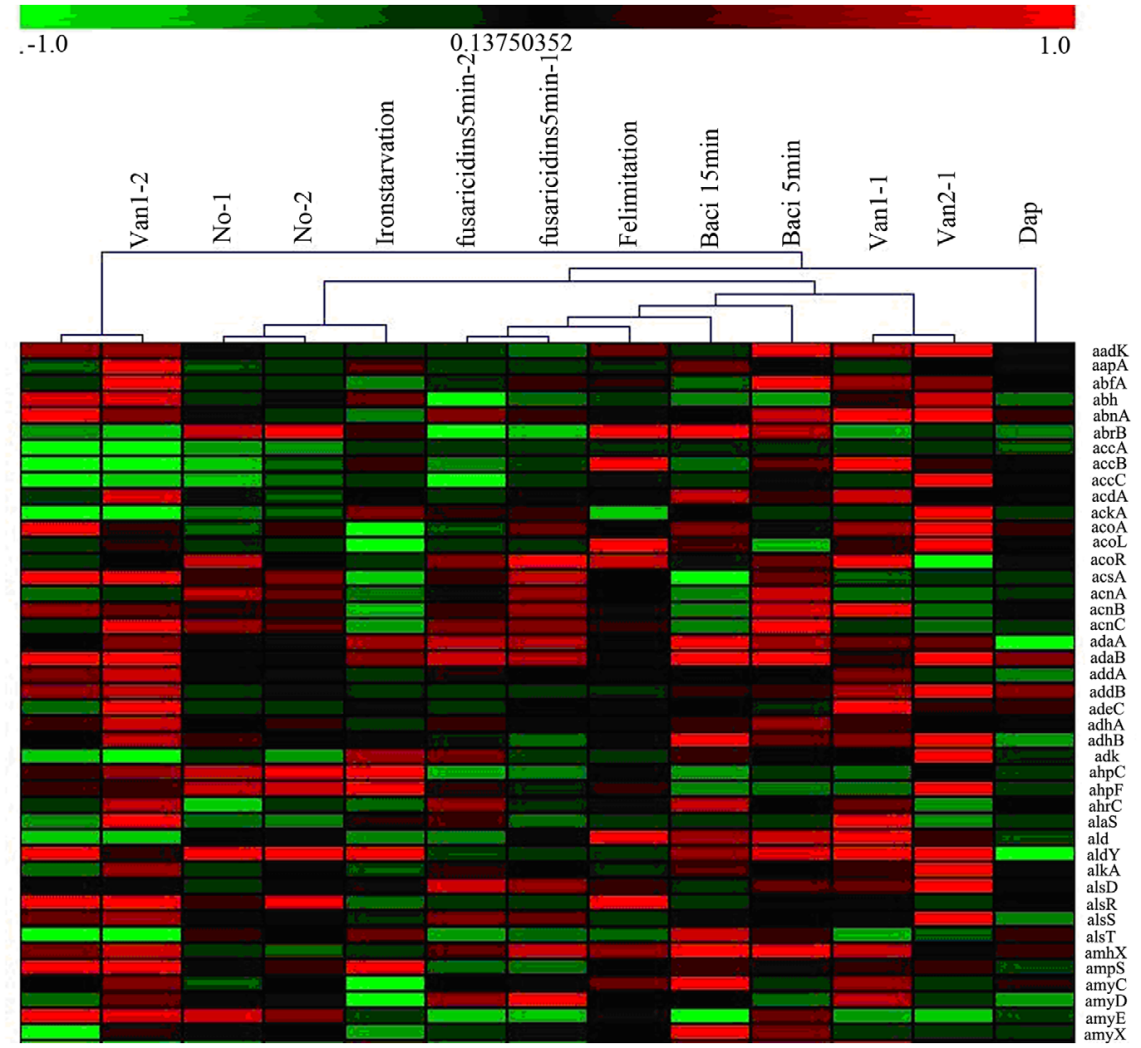
Clustering analysis of 6 experiments. Six individual experiments are listed on the top of the figure, and the names of the genes are shown on the right. The similarities of the genes between the different experiments are indicated in different colors. Low expression is indicated in green; and high expression, in red.

**Figure 9:**
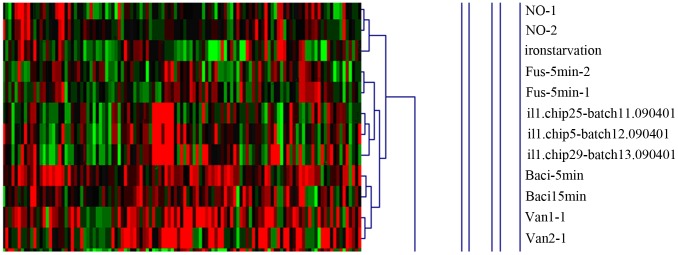
The clustering analysis between the antibiotic microarray data. Different antibiotics are listed on the top of the figure. The similarities of the genes between the different experiments are indicated in different colors. Low expression is indicated in green; and high expression, in red.

The function of differentially expressed genes could be divided into 2 categories: one is involved in the function of cell membrane (*yceD*, *ymcC*, *yuaFG*, *ythP*, and *yojB*), and the other is mainly related to detoxification, multidrug resistance, and cell protection (*yceE*, *ydjP*, and *yeaA*). *yceD* is involved in biofilm formation and was overexpressed by 3-fold after fusaricidin treatment, suggesting that accelerated biofilm formation may contribute to the resistance to toxins [14]. In *Escherichia coli*, the methionine sulfoxide reductase YeaA has an important function in protecting cells from oxidative damage [15]. It acts on free methionine sulfoxide (MetSO) and proteins that contain MetSO residues. Phenotypic analysis of an *E. coli* strain lacking a functional copy of *msrB* revealed its importance in cadmium resistance. Cadmium is a potential carcinogen and damages cells in several ways, including via the catalysis of AOS production [16]. YmcC is considered to be a lipoprotein and may therefore contribute to the membrane protection [17].

Most of the genes that were altered 5 min after the fusaricidin addition are involved in detoxification. The relationship among these rapid-response genes was determined using string analysis and is shown in Figure 2. *ybdK-ybdJ*, *kinA-spo0F*, *kinB-spo0F*, and *kinC* were closely correlated with the rapid-response phase. *kinA-spo0F* and *kinB-spo0F* are functionally important for bacterial spore formation. KinC is suggested to regulate gene expression during the stable phase, whereas the function of YbdK-YbdJ is currently unknown. As shown in Figure 2, KinB-Spo0F did not affect YdjPQ and YuaFGI directly, but KapB may function as an intermediate between them. The transmembrane protein YuaF from *B. subtilis* is a member of the NfeD-like clan with a potential role in maintaining membrane integrity during conditions of cellular stress [18]. We constructed a pathway of the rapid response phase, which incorporated gene expression and protein-protein interaction data (Fig. 3). It appears that the genes involved belong to a 2-component system (TCS). The TCS that is activated in response to fusaricidin includes modules involved in cellular membrane dynamics, as well as phosphorylation and dephosphorylation events associated with detoxification.

By the string analysis of pairwise combinations of TCS and kinases, we found that YbdK-YbdJ, KinA-Spo0F, KinB-Spo0F, and KinC were closely correlated with the rapid-response phase (5 min post treatment; see Fig. 2). The analysis also revealed that YdjPQ and YuaFGI were close to KapB, and KapB anchored with KinB, indicating that fusaricidins acted on the 2 TCS, KinB-SpoF and YbdK-YbdJ. In the next step, YbdK-YbdJ was activated by fusaricidin, and transcription of the genes downstream of this operon (*yvlA*, *yvlB*, *ymcC*, *pksA*, and *yeaA*) was significantly altered. The second TCS, KinB-SpoOF, was also induced by the fusaricidin treatment, changing the transcription of some of the downstream genes (*yvlA*, *yvlB*, *ymcC*, and *pksA*) involved in cell membrane dynamics. Furthermore, we found alterations in the other genes, although the precise biological implication of this remains unclear. It is possible that some of these genes modulate bacterial aggregation and/or growth.

### Effect of Fusaricidins on Carbon and Nitrogen Metabolism

Fusaricidin exposure for 20 and 170 min led to the induction of 194 genes by at least 3-fold, and many of these genes are members of known antibiotic-responsive stimulons (Table S1). A prominent feature was that a high proportion of these genes are regulated by *SigA*, which encodes the primary σ factor of RNA polymerase and is essential for cell growth. This result is in agreement with other studies on antibiotic treatment in *B. subtilis*, as there are some genes known to be induced by different antibiotics, such as *yvgN*, *ywiE*, *pyrB*, and *purC*. Although some characterized enzymes were present, including phosphoribosylglycinamide synthetase, many of the other genes encode proteins with no known function. The *dlt* operon, including *dltA*, is involved in the d-alanine esterification of lipoteichoic and wall teichoic acids, which increases bacterial resistance to cationic antimicrobial peptides. In this study, *dltA* was induced by more than 3-fold at 20 and 170 min (3.7- and 3.0-fold, respectively; Table S1), indicating that fusaricidin likely damages the cell wall and, in response, *B. subtilis* induces *dltA*. According to the MIPS analysis, the genes altered are mainly involved in glycolysis, the TCA cycle, and amino acid and fatty acid metabolisms (Fig. 4). CggR, YqzB, and YsiA were also significantly changed by the fusaricidin treatment. CggR modulates glucose catabolism, and it controls the expression of *pgm*, *gapA*, *pgk*, *tpiA*, and *eno*. *pckA*, *sped*, and *gapB*, all under the control of YqzB, were significantly repressed. Meanwhile, the genes regulated by the protein YsiA were overexpressed at 20 min, indicating that fusaricidin treatment may increase the degradation of fatty acids and histidine. As shown in Figure 4, the fusaricidin treatment increased the catabolism of fatty acids and amino acids but strongly repressed glucose decomposition and gluconeogenesis. This phenomenon indicates that the strains require increased energy to mount defenses against antibiotic peptides. It is generally thought that if the culture medium lacks sugars, amino acids are then broken down to provide the required carbon resource for other metabolic activities and gluconeogenesis should be concomitantly stimulated. However, our results appear to contrast with this hypothesis. According to the research of Ludwig [19] and Rezacova [20], CggR appears to respond synergistically to 2 different signals, one being the catabolic signal derived from the presence of sugars and the other being an anabolic signal derived from amino acid metabolism. The presence of the individual signals results in partial derepression of the *gapA* operon, whereas full induction occurs only if both signals are present. When glucose is absent from the growth medium, CggR binds to its target DNA sequence and blocks the transcription of genes in the *gapA* operon. In the presence of glucose, binding of fructose-1,6-bisphosphate abolishes this interaction, consistent with our previous observations [9]. Increased CggR activity leads to amino acid degradation, which leads to a reduction in the activity of *gapA*, *pgk*, *tpiA*, and *eno*. Therefore, we suggest that derepression of gluconeogenesis is a mechanism by which additional energy can be provided to mount a response to fusaricidin.

The MIPS analysis revealed significant changes in the genes involved in nucleotide metabolism (Table 1). The genes involved in the nucleotide metabolism pathway are shown in Figure 5, and the result of the pathway analysis indicates that the synthesis of purines and pyrimidines is repressed at an early stage. Nucleotide precursor degradation was increased by the fusaricidin treatment, indicating that the antibiotic likely reduced the availability of nucleic acid-related substances in *B. subtilis*.

Transcriptional factors play a central role in the restructuring of the transcriptome in response to environmental signals. The microarray data were subsequently analyzed using the T-profiler to identify the transcriptional factors that mediated the response to fusaricidin. The T-profiler is a computational tool that uses the *t* test to score changes in the average activity of predefined groups of genes based on the Gene Ontology categorization, upstream matches to a consensus transcription factor-binding motif, or the KEGG pathway [9]. In this study, the gene groups with significant *t* values (*E* = 0.05, TF model) are also presented in Table 2. Nine coregulated gene groups were found to be significantly perturbed by fusaricidin after 5 min, including SigW-, CcpA-, SigK-, SigE-, AbrB-, GerE-, FNR-, and SigB-regulated gene groups. As mentioned earlier, SigW probably activates a large stationary-phase regulon that functions in detoxification, production of antimicrobial compounds, or both. SigE and SigK regulate early and late mother cell-specific gene expression, respectively. AbrB is the regulon of transition state genes (negative regulation of *abrB*, *aprE*, *ftsAZ*, *kinC*, *motAB*, *nprE*, *pbpE*, *rbs*, *spoOH*, *spoVG*, *tycA*, *sbo-alb*, and *yqxM-sipW-tasA*, and positive regulation of *comK* and *hpr*).

Eleven and 13 gene groups were significantly modulated after 20 and 170 min of treatment, respectively. The results showed a strong activation of genes in the SigB regulon after the fusaricidin treatment. SigB is a general stress-response regulator that controls at least 150 genes. Members of the SigB regulon are transiently induced after heat shock or salt, ethanol, or acid stress, or upon limitation of glucose and phosphate starvation. In our study, a significantly negative *t* value for SigB was observed, which revealed that the fusaricidin addition repressed the expression of some SigB regulon genes (Table 2). CcpA is a global regulator of carbon metabolism in *B. subtilis* and mediates carbon metabolite repression [21]. The *t* values of the genes of the CcpA-negative group indicated that these genes were significantly overexpressed and that fusaricidin perturbs glucose metabolism. In *B. subtilis*, iron homeostasis is regulated by the ferric uptake regulator (Fur), which represses the expression of genes related to siderophore biosynthesis and iron uptake proteins. Iron limitation and oxidative stress are known to induce the Fur regulon [22]. The *t* values of the Fur-negative genes showed that this gene group were overexpressed. The StrCon-negative genes are involved in energy production, and the negative *t* values associated with this group indicate that the associated genes are somewhat overexpressed.

### Effect of Fusaricidins on Cation Transport

Fusaricidins had detrimental effects on the cell membrane, which would engender a loss of intracellular ions. This would lead to the induction of genes involved in ion uptake to maintain cell osmotic pressure and intracellular steady state. We studied the cation transport of *B. subtilis* after the addition of fusaricidin and observed that some genes involved in cation transport were significantly affected (Fig. 6).

Zinc is an important cofactor of many enzymes and for protein folding and is transported by 3 uptake systems, *yciABC*, *ycdHI-yceA*, and *zosA*(*ykvw*). *yciABC* is regulated by Zur, which was the negative regulator of zinc uptake. In *B. subtilis*, the genetic response to zinc starvation included, as expected, the derepression of a high-affinity zinc uptake system and a high-affinity zinc ABC transporter encoded by the *ycdHI-yceA* operon [23]. *zosA* is regulated by PerR, the peroxide sensing repressor, and is not inhibited by Zn^2+^. Zur also represses 3 genes (*ytiA*, *rpmGC*, and *yhzA*) that encode paralogs of ribosomal proteins [24]. The *ytiA* gene encodes an alternative form of L31 that lacks zinc. L31, encoded by *rpmE*, is a small, zinc-containing protein that is associated with the large ribosomal subunit [25]. When zinc is limiting in the cell, YtiA is expressed, causing the displacement of L31 (RpmE) from the ribosome. This is thought to liberate zinc for essential cellular functions. Meanwhile, the *B. subtilis* Zur protein repressed the expressions of at least 10 genes in response to zinc. In our study, *yciC*, *ycdH*, and *yceA*, which are all involved in zinc transport, were upregulated. Concomitantly, we observed an upregulation of *rpmC* and *yhzA*. The above-mentioned results indicate that cells require more zinc to mount a defense against fusaricidin damage.

The transport and oxidation stress response associated with ferrous ion and manganous are shown in Figure 7. The formation of intracellular reactive oxygen species (ROS) is potentially a byproduct of metabolism after fusaricidin treatment in an aerobic environment. Microorganisms have evolved an impressive array of mechanisms to adapt to stress induced by virtually all types of ROS. One such regulator is PerR, a member of the ubiquitous Fur family of metalloregulatory repressors, which sense hydrogen peroxide. PerR uses a metal, Fe(II) or Mn(II), to activate operator DNA binding; however, PerR cannot bind Fe(II) or Mn(II) when H_2_O_2_ is present. Zn(II)-bound PerR appears to replace the Fe(II)- or Mn(II)-bound species, which can lead to an increase in *mrgA*, *katA*, and *ahpCF* [26]. According to the speculation of Fuangthong [27] and Herbig [28], the inhibition of Mn(II) transport may be a way for cells to protect themselves. Sufficiently high concentrations of Mn(II) lead to significant PerR inhibition, which remains unaffected by the presence of peroxide. This would essentially prevent the induction of detoxification genes and limit the cell’s ability to mount a defense. However, when the Fe(II) concentration was gradually reduced, PerR activity in response to peroxide was restored. In *B. subtilis*, iron is transported through 3 steps: (1) threonine, glycine, and 2,3-dihydroxybenzoate are used as precursors to synthesize bacillibactin (BB) by *dhbCAEBF*; (2) BB is then exported from the cell by YmfE to combine with iron; and (3) Fe-BB is shuttled back into the cell via the ABC-type transporter FeuABC-YusV. To achieve intracellular iron release, Fe-BB is then hydrolyzed by the Fe-BB esterase BesA and iron is used by the cell [27]. The process of iron transport is controlled by 3 regulatory proteins: Fur, Mta, and Btr. When iron concentration is low, derepression of Fur leads to increased activity of Mta and Btr, which accelerates BB outflow and Fe-BB uptake. In this manner, all the genes related to iron transport are upregulated upon fusaricidin treatment of *B. subtilis*, robustly stimulating iron transport.

We next compared our data with the results from other studies. Cluster analysis was used to determine whether other antibiotic treatments had a similar profile to that of fusaricidin. NO [28], vancomycin (Van) [18], bacitracin (Baci) [29], iron starvation [30], Fe limitation [31], and daptomycin (Dap) [32] were all used in the comparison. As shown in Figure 8, the data from the Fe limitation treatment had the highest similarity to those from our experiment. This suggests that iron is an essential component for bacteria to resist treatment with toxins. Forty additional antibiotics were also chosen to compare with the fusaricidin treatment in this study. This comparison revealed that the treatment of *B. subtilis* with fusaricidin elicited a profile most similar with that of triclosan (Fig. 9).

Fusaricidin addition could lead *B. subtilis*’s membrane to be destroyed and more OH produced which affected the biosynthesis of protein and nucleic acid in the cells at the initial phase. However, *B. subtilis* could recover its growth in the late phase because of the congeries of the cells in the culture (data not shown here). It is suggested that the novel antibactin should stimulate the cells to secrete more and more OH to disturb the growth and prevent the cells to congest simultaneously.

The transcriptome analyses indicate that fusaricidin induced sets of genes shown previously to be induced by exposure to membrane-active compounds. The TCS was significantly induced by fusaricidin, and genetic studies indicated that SigA was sensitive to this change. These results were consistent with the notion that this type of antibiotic acts primarily on the cell membrane [33]. Apparently, *B. subtilis* is one of microorganisms which is able to alter its gene expression pattern in response to fusaricidin to develop resistance to antibiotic treatment and some other environmental changing.

## Supporting Information

Table S1 **Gene Differentially expressed genes at 20 and 170 min.**
(XLSX)
